# Health-promotion can sustain leisure participation among persons 80 years and older: results from the randomised controlled trial elderly persons in the risk zone

**DOI:** 10.1186/s12877-026-07401-1

**Published:** 2026-04-01

**Authors:** Roar Hermansen Østby, Synneve Dahlin-Ivanoff, Jenna Najar, Qarin Lood

**Affiliations:** 1 University of Gothenburg, Box 455, Gothenburg, SE- 40530 Sweden; 2 University of Gothenburg Centre for Person-Centred Care (GPCC), Sahlgrenska Academy, University of Gothenburg, Gothenburg, Sweden; 3 Department of Psychiatry and Neurochemistry, Institute of Neuroscience and Physiology, Sahlgrenska Academy, University of Gothenburg, Blå stråket 15, vån 3 SU/Sahlgrenska, Gothenburg, SE-413 45 Sweden; 4 Alzheimer Center Amsterdam, Neurology, Vrije Universiteit Amsterdam, Amsterdam UMC Location VUmc, Amsterdam, The Netherlands; 5 Amsterdam Neuroscience, Neurodegeneration, Amsterdam, The Netherlands

**Keywords:** Health-promotion, Hobbies, Intervention, Leisure activities, Municipal care, Older adults, Prevention, Recreation

## Abstract

**Background:**

Participation in leisure activities is associated with better health, greater life satisfaction, and improved quality of life in older persons, yet it tends to decline with age. Although health-promotion interventions positively influence such participation, evidence is limited for persons aged 80 years and older. This study aimed to examine whether a preventive home visit or senior meetings affect participation in leisure activities among community-dwelling persons aged 80 years or older.

**Methods:**

The study was conducted in Gothenburg, Sweden, applying an exploratory, randomised, three-armed, single-blind, controlled trial with a parallel-group design. Participants were 459 independent, community-dwelling persons aged 80 years or older, randomly assigned to one of two interventions or an inert control group using opaque envelopes. A total of 178 participants were randomised to the preventive home visit group, 199 to the senior meetings group, and 114 to the control group. Participants analysed were 174, 171, and 114 respectively. Interventions included either a preventive home visit or senior meetings. The primary outcome was participation in leisure activities at 12- and 24-months post-baseline. Outcome assessors were blinded after assignment.

**Results:**

At 12-months, the senior meetings group sustained participation in senior activities (OR 1.77, 95% CI 1.05–2.99), reading (OR 1.83, 95% CI 1.03–3.25), and hobbies (OR 1.95, 95% CI 1.14–3.33), compared with controls. The preventive home visit group sustained participation in hobbies (OR 1.74, 95% CI 1.03–2.94), compared with controls. At 24-months, the senior meetings group sustained participation in study circles (OR 1.99, 95% CI 1.09–3.66), reading (OR 1.79, 95% CI 1.07-3.00) and hobbies (OR 1.87, 95% CI 1.12–3.12), compared with controls. The preventive home visit group sustained participation in reading (OR 2.09, 95% CI 1.24–3.52), compared with controls. No adverse effects were reported.

**Conclusions:**

Senior meetings supported sustained participation in several leisure activities among persons aged 80 and older over 24 months. Tailoring support to individual needs and adapting to changing abilities may enhance long-term participation and well-being. Key elements of effective interventions may include providing information, fostering positive attitudes, and encouraging open dialogue about leisure.

**Trial registration:**

The study was registered 08/04/2009 at ClinicalTrials.gov, registration number NCT00877058.

## Background

Participation in leisure activities has been linked to numerous positive outcomes for older persons, including improved physical and mental health, life satisfaction, and overall quality of life [1–5]. Additionally, participating in such activities can help reduce the risk of developing dementia over time, although robust experimental evidence remains limited [6]. Defined as freely chosen pursuits that bring enjoyment [7], leisure activities often increase following retirement from paid work [3]. However, Nilsson et al. [8] describe a negative trend across the ageing span, with a significant decrease in participation in leisure activities between the ages 65 and 80. Thus, as people age, their participation in leisure activities tends to decrease [8], but little is known about how to promote participation in leisure activities in old age.

According to the latest statistics (2025), there are 684,378 persons aged 80 years or older living in Sweden, a number expected to rise to 1,067,889 by 2050 [9]. While a substantial proportion 80-year-olds enjoy good health and quality of life while living in the community, this group is often vulnerable to diseases, disabilities, and frailty [10–12]. Frail older persons are characterised by reduced resilience and resistance to stressors due to cumulative physiological decline, leading to increased vulnerability and functional deterioration [11]. Additionally, frailty is associated with activity limitations, participation restrictions, and comorbidities [10].

Earlier studies show that health-promotion interventions performed before the onset of frailty can slow down the decline in self-rated health and postpone both dependence in activities of daily living [13] and frailty [14]. Research has also demonstrated that engaging in leisure activities improves health by lowering stress, increasing social support [15] and maintaining cognitive, physical, and mental health [16, 17]. The multi-level leisure mechanisms framework identifies four main processes through which leisure activities may affect health:

1. Psychological processes: building resilience and supporting a sense of self.
2. Biological processes: Enhancing various aspects of physical performance.
3. Social processes: Improving social relations.
4. Behavioural processes: Increasing habits and motivation [18].

Moreover, Yang et al. [19] recently reported that interventions including leisure activities positively affect the postponement of frailty in older persons. Understanding how to promote participation in leisure activities among older persons is therefore crucial for effective health-promotion and proactive care practices. Health-promotion and proactive care is receiving increasing support in healthcare policy; for example, Sweden is transitioning towards more proactive, health-promoting, and person-centred healthcare and social services across organisations [20], and the World Health Organization emphasises health-promotion and disease prevention as central components of a comprehensive healthcare approach [21]. These current reforms highlight the importance of addressing multiple health determinants within the interplay between persons, their daily activities, and their environments.

Drawing on Wilcock and Hocking’s occupational perspective of health [22], which conceptualises engagement in everyday activities (occupations) as fundamental to human existence and as a key determinant of health, this study builds on the premise that participation in everyday activities are central to health and well-being. Within this perspective, occupation encompasses dimensions of active engagement in purposeful activities (doing), experiencing meaning and identity (being), growth and development over time (becoming), and social connectedness (belonging). Together, these dimensions illustrate how participation in everyday activities contribute to the development and maintenance of health resources [22]. This perspective is particularly relevant in efforts to prevent frailty and support independence in later life.

The study is part of the randomised controlled trial (RCT) called “Elderly persons in the risk zone”, which compared the effects of a preventive home visit and multiprofessional, multidimensional senior group meetings among community-dwelling persons aged 80 years or older who were at risk of developing frailty [23]. Within this context, healt-promotion integrates both health improvement and prevention, aiming to support positive health outcomes while also preventing the progression of frailty, illness, and functional decline. The interventions were developed using a multiprofessional and participatory approach. The aim of the present study was to examine whether a preventive home visit or senior group meetings influence participation in leisure activities among community-dwelling persons aged 80 years or older. We hypothesised that when health-promotion interventions are implemented while older persons are still living independently in the community, participation in leisure activities can be sustained over time.

## Methods

### Participants and setting

The study was conducted in Gothenburg, Sweden. To be eligible for participation in the trial, participants had to be 80 years or older and living in ordinary housing, defined as private homes or apartments (i.e., not in residential care facilities). Participants were also required to not be dependent on municipal home help services or formal care, be independent in daily activities, and to have a Mini Mental State Examination (MMSE) score of 25 or higher. These strict inclusion criteria were chosen to ensure a relatively homogeneous and initially independent population, enabling the study of changes over time without results being substantially influenced by advanced comorbidities or functional impairments, and reducing potential confounding factors related to disease progression rather than the intervention. To achieve a representative sample, eligible participants were identified through official population registers including all persons 80 years or older in two urban districts in Gothenburg. At the time of the trial, persons over 80 years of age constituted 8% and 7% of the population in the respective districts, compared with 5% in Gothenburg and Sweden overall. The districts were located within city limits but outside the city centre and consisted of a mix of privately owned houses and apartment buildings. Educational and income levels were slightly higher, and illness rates somewhat lower, than in the general Gothenburg population. Equal numbers of persons from the two districts were listed in random order and included consecutively using simple random sampling until the desired sample size was reached.

### Sample size

The power calculation was based on the anticipated difference in the change in functional abilities over time between the study groups (alpha level of 0.05 and a power of 80% for a two-sided test). Consequently, at least 112 participants were needed in each intervention group to detect a minimum difference of 15% between the groups. For comparisons between the control group and the intervention groups, 72 participants were required in the control group, assuming a difference of at least 20%. Therefore, it was determined that a minimum of 300 participants was necessary, and 459 participants were included to account for potential dropouts.

### Study design

Elderly persons in the risk zone was an exploratory, randomised, three-armed, single-blind, controlled trial with a parallel-group design. The trial had two intervention groups and one control group. Follow-up assessments were conducted at three, 12 and 24 months [23]. The trial was conducted between November 2007 and March 2011. Since this study focuses on the sustainability of effects, this report only includes data from the 12- and 24-months follow-ups. The regional Ethical Review Board in Gothenburg approved the study (ref.nr: 650-07). Written informed consent was obtained from the participants before study commencement and the study was registered at Clinical Trials (registration number NCT00877058). This report followed the guidelines outlined in the CONSORT statement for reporting RCTs ([24], See Additional file 1).

### Interventions

The study comprised two intervention groups, preventive home visit or senior meetings, and one inert control group. The interventions were developed through a multiprofessional and participatory process. Research and project teams were established, comprising researchers and health and social care professionals to facilitate collaboration throughout the development and implementation phases. This approach enabled integration of evidence-based knowledge with professional experience and local contextual factors [25, 26]. Active involvement of older persons was a guiding principle, consistent with participatory research approaches [27]. Following consultations with a local pensioners’ association, a reference group was formed at an early stage of the project. The group remained involved throughout the study period and participated in regular meetings within the “Elderly persons in the risk zone” trial. The reference group contributed to intervention design, testing of instruments, and a review of study materials.

#### Preventive home visit

The intervention involved a single home visit by a registered nurse, physiotherapist, social worker, or occupational therapist. During the visit, participants received verbal and written information about local meeting places, activities, physical training for seniors, walking groups, and assistance provided by volunteers or professionals. They were informed about assistive devices, housing adaptations, fall risk identification, and prevention strategies. Additionally, participants were given contact information for various problems.

#### Senior meetings

The intervention consisted of four weekly, two-hour educational senior meetings, each involving up to six participants. The primary objectives were to provide information about and discuss the ageing process and its consequences, and to offer tools and strategies for addressing various problems in the home environment. A follow-up home visit was conducted two to three weeks after the final group meeting. Each meeting was facilitated by health and social care professionals, including an occupational therapist, physiotherapist, registered nurse, physiotherapist, and a social worker, each responsible for education content relevant to their area of expertise. A specially designed booklet, containing self-care strategies and supplementary information aligned with the meeting topics, served as a key educational resource.

#### Control group

Participants in the control group had access to the standard range of services provided by municipal aged care. If the investigator identified any needs among the control group members, they were informed about the appropriate municipal services to address their issues.

For a more detailed description of the interventions, see the study protocol for “Elderly persons in the risk zone” [23].

### Procedure

Eligible persons aged 80 years or older were identified from official registries in the two included districts. Equal numbers from each district were randomly ordered, and participants were selected consecutively using simple random sampling until the target sample size of 2031 was reached. Invitation letters describing the study and emphasising voluntary participation were mailed, followed by a phone call one to two weeks later.

Of the 2031 persons, 365 were ineligible or untraceable, leaving 1666 contacted. After receiving oral information, 1120 declined (no interest = 936, lack of time = 116, insufficient strength = 68). The remaining 546 persons provided informed consent and completed baseline home interviews conducted by trained research assistants, who also performed follow-up assessments. Outcome assessors were blinded to group assignment and not involved in the intervention.

**Fig. 1:**
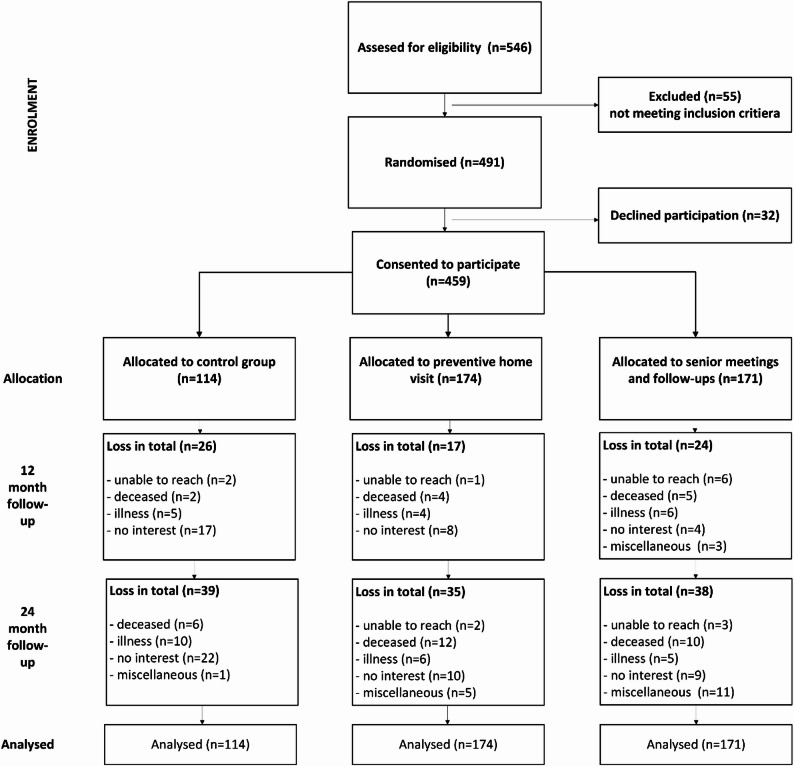
The flow of participants through the study, including reasons for dropout at 12 and 24 months

Of these 546, 491 met the inclusion criteria and were randomised: 178 to the preventive home visit group, 199 to the senior meetings group, and 114 to the control group. A total of 32 persons declined participation within their assigned interventions (5 from home visit, 23 from senior meetings, and 5 due to illness), leaving 459 participants in the study: 174 in the preventive home visit group, 171 in the senior meetings group, and 114 in the control group (Fig. 1).

### Data collection and outcome measure

Data collection for the study was conducted by research assistants, including occupational therapists, physical therapists, and registered nurses, who visited all participants in their homes at baseline, and three follow-ups (three, 12 and 24 months). The visits included a structured interview, formal assessments, and observations to collect data on demographic characteristics (age, gender, educational level, self-rated health, living conditions, i.e., living alone), frailty, activities of daily living, quality of life, life satisfaction, assistive technology, accessibility, feeling of loneliness, social interaction, social support, leisure activities, falls, fear of falling, healthcare consumption, and mortality. The interview was not developed specifically for this study but is part of several intervention studies within the FRESH research programme *“Ageing Well at Home – Measures for Supporting Frail Older People”*, compiled in the FRESH database. The study is registered at ClinicalTrials.gov (NCT00877058) and the database protocol is preregistered in the Open Science Framework (OSF) ([10.17605/OSF.IO/T9K7Q]) to ensure transparency and reproducibility.

All research assistants received specific training on administering the interview, assessments, and observations. Interrater reliability was evaluated and maintained through group discussions to ensure consistency. To maximise standardisation, regular study protocol meetings were held throughout the trial to maintain uniformity in assessments. Data on home-help services and mortality were obtained from municipal records. The outcome of interest for this study was change in leisure participation at the 12- and 24-months follow-ups.

For this outcome, we took inspiration from earlier studies about leisure activities, among them the Modified NPS (Norling, Pettersson, Selander) Interest Checklist (MNPS) [28], but also the work of Silverstein and Parker [29] and Iso-Ahola et al. [30]. From these works we derived four leisure categories: Cultural, Social, Physical, and Hobbies/other activities with two leisure activities in each category, namely:

Cultural activities.

- Going to the movies, attending concerts, visiting museums or exhibitions.
- Church activities.

Physical activities.

- Gymnastics, swimming or cycling.
- Light physical exercises such as boules or walking groups.

Social activities.

- Senior activities.
- Study circles and courses.

Hobbies/other activities.

- Reading books, newspapers, or magazines.
- Hobbies, such as knitting, sewing, woodworking, painting, or other similar activities.

Participants were assessed through structured interviews to answer whether they participated in the activities: *Yes*,* often / Yes*,* sometimes / No*.

### Statistical methods

All analyses were conducted based on the intention-to-treat (ITT) principle [31]. The premise for data imputation was that persons aged 80 years or older were expected to deteriorate over time as part of the natural ageing process. The ITT principle, primarily used in RCT analyses, includes all participants in their originally assigned groups, regardless of study completion, adherence to the treatment protocol, or attrition. This approach preserves the original randomisation and prevents potential bias [32], providing a fair comparison between intervention groups and reflecting treatment-policy effects rather than specific intervention effects [23].

The Median Change of Deterioration (MCD) for each leisure activity was calculated and used to impute missing values [33]. Missing values due to death were imputed using worst-case values at each follow-up [34]. The MCD method estimates missing values based on the median change observed between measurement points. For participants with available data at both baseline and follow-up (12 and 24 months), the individual change in values was calculated, and the median of these changes was used to impute missing values at the corresponding follow-up.

The MCD approach was selected because participants who dropped out had significantly poorer baseline values, suggesting that the missing data mechanism was unlikely to be missing at random. Under such conditions, model-based multiple imputation may rely on assumptions that are difficult to justify and may introduce bias. In contrast, MCD provides a transparent, non-parametric imputation strategy that reflects the expected gradual decline typically observed among persons aged 80 years and older. The approach is conservative because it is more likely to underestimate than overestimate intervention effects. Sensitivity analyses comparing MCD with complete case analyses were conducted.

Baseline and dropout characteristics among the three groups were compared using Chi-square tests or Fisher’s exact test for dichotomous variables, Mann–Whitney U test for ordinal data, and t-test for continuous variables. The outcome measure participation in leisure activities was dichotomised into sustained (Yes, often/Yes, sometimes) or non-sustained (No), analysed using a Chi-square test, followed by group-wise comparisons using odds ratios (OR). Two-sided significance tests were consistently applied to ensure the robustness of the results. A p-value < 0.05 was considered statistically significant. All statistical analyses were performed using IBM SPSS Statistics Version: 29.0.0.0. Because several pre-specified leisure outcomes were analysed across two intervention groups and two follow-up time points, the issue of multiple comparisons was considered. As the analyses were exploratory and aimed to examine patterns of sustained leisure participation rather than to test a single confirmatory hypothesis, no formal adjustment for multiple comparisons was applied.

## Results

The median age of participants was 86 years (range 80–94 years) in preventive home visit, 85 years (range 80–94 years) in senior meetings and 86 years (range 80–97 years) in the control group. Dropout rates in the control group were higher than in the intervention groups, see Table 1 for an overview of dropout rates across all groups.

**Table 1:** Overview of dropout rates

Group	12 months	24 months
Overall	23%	25%
Preventive home visit	10%	21%
Senior meetings	14%	23%
Control	23%	34%

There were no statistically significant differences across groups in terms of demographic characteristics at baseline. Most of the participants rated their health as excellent, very good, or good (Table 2).

**Table 2:** Demographic characteristics of study participants at baseline

Characteristics	Control group *n* = 114	Preventive home visit *n* = 174	Senior meetings *n* = 171	
	%	%	%	*P*-value
Female	61	64	66	0.63
Living alone	48	57	60	0.10
Academic education	22	23	19	0.69
Self-rated health (excellent/very good/good)	79	80	83	0.63

The interventions were delivered as planned. All participants assigned to preventive home visit received it. Of those in the senior meetings, 97% attended all four sessions, 2% attended three, and 1% attended two. No adverse events or known co-interventions were identified during the study period.

Independent of group allocation, participants were mostly active in cultural activities and hobbies/other activities, while participants were less active in physical activities (Table 3).

**Table 3:** Distribution of participants’ participation in the measured leisure activities across groups at baseline and follow-ups

	Group	Baseline *n* (%)	12 months *n* (%)	24 months *n* (%)
**Cultural activities**				
Going to the movies, concerts etc.	Control	78 (68)	50 (44)	37 (33)
	Preventive home visit	114 (66)	97 (56)	79 (45)
	Senior meetings	118 (69)	93 (54)	83 (49)
Church activities	Control	20 (18)	18 (16)	17 (15)
	Preventive home visit	41 (34)	44 (35)	43 (25)
	Senior meetings	34 (20)	39 (23)	32 (19)
**Physical activities**				
Gymnastics, swimming, cycling etc.	Control	25 (22)	21 (18)	11 (10)
	Preventive home visit	37 (21)	42 (24)	27 (16)
	Senior meetings	35 (21)	39 (23)	16 (9)
Light exercise, boules, walking, etc.	Control	19 (17)	17 (15)	13 (11)
	Preventive home visit	30 (17)	26 (15)	16 (9)
	Senior meetings	22 (13)	22 (13)	16 (9)
**Social activities**				
Senior activities	Control	52 (46)	33 (29)	31 (27)
	Preventive home visit	86 (49)	70 (40)	68 (39)
	Senior meetings	65 (38)	73 (43)	60 (35
Study circles and courses	Control	29 (25)	14 (12)	9 (8)
	Preventive home visit	37 (21)	24 (14)	19 (11)
	Senior meetings	27 (16)	35 (21)	27 (16)
Hobbies/other activities				
Reading books/newspapers/ magazines	Control	109 (96)	85 (75)	74 (65)
	Preventive home visit	172 (99)	149 (86)	160 (92)
	Senior meetings	169 (99)	164 (96)	159 (93)
Hobbies, such as knitting, sewing, woodworking, painting etc.	Control	53 (47)	38 (33)	23 (20)
	Preventive home visit	71 (41)	58 (33)	59 (35)
	Senior meetings	59 (35)	72 (42)	65 (38)

At the 12-month follow-up, the analysis showed statistically significant differences in favour for the intervention group senior meeting compared to the control group in: senior activities (OR 1.77, 95% CI 1.05–2.99), Reading books/newspapers/magazines (OR 1.83, 95% CI 1.03–3.25) and Hobbies, such as knitting, sewing, woodworking, painting, or other similar activities (OR 1.95, 95% CI 1.14–3.33). For the preventive home visit group, there were statistically significant differences compared to the control group in: Hobbies, such as knitting, sewing, woodworking, painting, or other similar activities (OR 1.74, 95%CI 1.03–2.94). See Table 4 for details.

**Table 4:** Sustained participation in leisure activities from baseline to 12 months

	Control group (*n* = 114)		Preventive home visit (*n* = 174)			Senior meetings (*n* = 171)		
	%	OR	%	OR (CI)	*P*-value	%	OR (CI)	*P*-value
**Cultural activities**								
Going to the movies. concerts etc.	56	1	67	1.60 (0.99–2.61)	0.06	63	1.31 (0.81–2.12)	0.28
Church activities	89	1	84	0.67 (0.33–1.36)	0.27	86	0.79 (0.38–1.62)	0.52
**Physical activities**								
Gymnastics, swimming, cycling etc.	82	1	83	1.06 (0.57–1.99)	0.85	84	1.13 (0.60–2.14)	0.70
Light exercise, boules, walking etc.	85	1	86	1.04 (0.54–2.04)	0.90	87	1.19 (0.60–2.35)	0.62
**Social activities**								
Senior activities	65	1	68	1.14 (0,69-1.88)	0.61	77	1.77 (1.05–2.99)	**0.03**
Study circles and courses	76	1	81	1.33 (0.75–2.36)	0.34	85	1.81 (0.99–3.32)	0.05
**Hobbies/other activities**								
Reading books/newspapers/magazines	73	1	82	1.66 (0.94–2.91)	0.08	83	1.83 (1.03–3.25)	**0.04**
Hobbies, such as knitting, sewing, woodworking, painting etc.	66	1	77	1.74 (1.03–2.94)	**0.04**	79	1.95 (1.14–3.33)	**0.01**

Bold p-values indicate statistically significant p-value ≤ 0.05

At the 24-month follow-up, the analysis showed statistically significant differences in favour for the intervention group senior meetings compared to the control group in: Study circles and courses (OR 1.99, 95% CI 1.09–3.66), Reading books, newspapers, or magazines (OR 1.79, 95% CI 1.07-3.00) and Hobbies, such as knitting, sewing, woodworking, painting, or other similar activities (OR 1.87, 95% CI 1.12–3.12). For the preventive home visit group, participation was sustained for Reading books, newspapers, or magazines (OR 2.09, 95% CI 1.24–3.52). See Table 5 for details.

**Table 5:** Sustained participation in leisure activities from baseline to 24 months

	Control group (*n* = 114)		Preventive home visit (*n* = 174)			Senior meetings (*n* = 171)		
	%	OR	%	OR (CI)	*P*-value	%	OR (CI)	*P*-value
**Cultural activities**								
Going to the movies, concerts etc.	50	1	59	1.42 (0.88–2.28)	0.15	58	1.38 (0.85–2.21)	0.19
Church activities	89	1	85	0.73 (0.36–1.49)	0.39	87	0.83 (0.40–1.71)	0.61
**Physical activities**								
Gymnastics, swimming, cycling etc.	82	1	82	1.00 (0.54–1.84)	1.00	82	1.06 (0.57–1.97)	0.85
Light exercise. boules, walking etc.	85	1	84	0.95 (0.49–1.84)	0.89	88	1.25 (0.63–2.49)	0.52
**Social activities**								
Senior activities	63	1	68	1.26 (0.77–2.07)	0.36	73	1.59 (0.95–2.64)	0.08
Study circles and courses	75	1	80	1.34 (0.76–2.37)	0.31	86	1.99 (1.09–3.66)	**0.03**
**Hobbies/other activities**								
Reading books/newspapers/magazines	63	1	78	2.09 (1.24–3.52)	**0.01**	75	1.79 (1.07-3.00)	**0.03**
Hobbies, such as knitting, sewing, woodworking, painting etc.	61	1	71	1.56 (0.95–2.57)	0.08	75	1.87 (1.12–3.12)	**0.02**

Bold p-values indicate statistically significant p-value ≤ 0.05

## Discussion

In this RCT, we evaluated the effects of two health-promotion interventions, i.e., a preventive home visit and senior meetings, on participation in leisure activities among community-dwelling persons aged 80 years and older. We hypothesised that early intervention, while older persons are still living independently in the community, could help sustain participation in leisure activities over time. The findings partly confirm this hypothesis, showing that both interventions could support participation in social activities and hobbies, but not in physical or cultural activities. The finding that senior meetings influenced a broader range of leisure activities than preventive home visit aligns with previous research indicating that multiprofessional group interventions may be more effective than single-session home visits [13, 14]. The group format of the senior meetings may have fostered peer learning, social connectedness and self-determination [35], key determinants of behavioural maintenance. Such group-based interventions may enhance motivation and capability and expand opportunity for participation [36], processes likely to explain why the senior meetings were more effective in sustaining overall leisure participation compared with preventive home visits and usual care. In the context of previous research, these findings are important as leisure activities have been shown to enhance psychological well-being, resilience, and cognitive health in later life. Previous research confirms that participation in leisure activities correlates positively to both mental health and preserved cognitive and physical function [16, 37, 38]. A recent meta-analysis also demonstrated that leisure interventions can significantly improve memory and executive functions among older persons [39]. Thus, health-promotion interventions, such as the senior meeting, which promote reflection, peer discussion, and self-determination in activity choices, may indirectly support long-term well-being and cognitive resilience. A possible explanation for the lack of intervention effects on cultural and physical activities could be accessibility and environmental barriers. Older persons may avoid activities requiring travel or substantial effort, such as attending the opera or cinema, due to declining mobility, transportation challenges, or cost. In contrast, home-based or nearby activities, like reading or crafts, may be more easily maintained. This interpretation aligns with a recent systematic review, identifying mobility limitations, safety concerns, and lack of suitable opportunities as major barriers to participation in physical and cultural activities among older persons [40]. Another consideration is that the present study did not capture whether leisure activities were chosen or performed for pleasure or personal meaning. Previous research has shown that intrinsic motivation, such as enjoyment, fulfilment, or a sense of purpose, is a strong determinant of sustained participation in physical leisure activities among older persons [41]. Without understanding the motivational or affective dimensions of leisure participation, it is difficult to discern whether participants continued activities out of genuine interest or habit, or whether they discontinued them because they no longer found them enjoyable or rewarding. This gap points to the importance of exploring not only what activities older persons participate in and to what extent, but why they do so. Future studies should therefore include measures of intrinsic motivation, satisfaction, engagement, and perceived meaning of leisure activities. Furthermore, some older persons may discontinue activities that they can no longer perform as before, either due to physical limitations or financial constraints, which can reduce motivation to re-engage [42]. This highlights the need for adaptive leisure strategies that modify activities to match changing capabilities. Recent evidence also suggests that interventions that promote personal challenge and social interaction can mitigate withdrawal from previously valued activities [41]. Together with the varying effectiveness of the two interventions, these findings underscore the importance of person-centred approaches in health-promotion, particularly in supporting the leisure activities that older persons value most. This is also in line with the global transition of healthcare and social service systems towards person-centred integrated care [43] and with person-centred care emphasising respect for individual preferences, shared decision-making, and partnership [44, 45]. Although not explicitly designed as person-centred, both interventions in this study reflected such principles through participatory development and flexible content [46]. The senior meetings, in particular, encouraged participants to discuss self-selected topics [23], supporting self-determination and participation in activities. This approach resonates with contemporary models of healthy ageing, which emphasise self-determination, meaningful activity, and social participation as core dimensions of well-being [47]. Importantly, person-centredness was embedded from the outset through the co-creation process: a reference group of older persons, identified in collaboration with a local pensioners’ association, was involved from the planning phase and remained engaged throughout the entire project. This group contributed to shaping the intervention content, testing instruments, and validating materials, ensuring that the programme aligned with the needs, preferences, and lived experiences of the target population. Such involvement reflects the essence of person-centred care, recognising older persons as active partners rather than passive recipients.

Future interventions could further integrate person-centred approaches by incorporating shared decision-making and goal setting, follow-up support, and environmental adaptations tailored to individual resources and needs, as these factors are central to sustained participation, particularly when physical or contextual barriers arise. Overall, these findings suggest that structured, interactive, and multiprofessional health-promotion interventions can play a valuable role in sustaining leisure participation in very old age. However, further research is needed to better understand the meaningfulness, motivation, and personal value of different leisure activities, as these factors are likely key to long-term participation and well-being. For policymakers and practitioners, integrating such comprehensive interventions into community-based care and service may help older persons maintain not only leisure participation, but also their physical, cognitive, and emotional health. Future initiatives should aim to combine person-centred approaches with environmental and structural supports to create systems that enable older persons to continue leading active, fulfilling, and meaningful lives.

### Strengths and limitations

One of the key strengths of this study is its rigorous design. The three-armed RCT design enabled direct comparisons between two active interventions and an inert control group, thereby strengthening the internal validity of the findings. Randomisation was successfully implemented, and no significant differences were observed in baseline characteristics across the groups. This supports the conclusion that observed differences in outcomes are likely attributable to the interventions themselves.

Prior to the main study, a pilot study was conducted to test the feasibility of the interventions, inclusion criteria, and logistics. This preparatory phase involved both pensioner representatives and municipal health and social care professionals, ensuring that the study design was grounded in the lived experiences and practical realities of the target population. Feedback from pilot interviews led to refinements in the outcome measures and interview protocols, thereby improving the clarity and relevance of the data collection instruments. Participant involvement was a key feature throughout the study. Older persons contributed to the planning and development of the interventions, including the educational content and materials. This participatory approach likely increased the acceptability and relevance of the interventions, potentially enhancing their effectiveness.

The study also benefited from a multidisciplinary research and implementation team comprising professionals from occupational therapy, physiotherapy, medicine, nursing, social work, and health economics. This diversity enabled a comprehensive approach to both intervention design and outcome assessment. Furthermore, outcome measures were clearly defined and validated, and data collection was conducted by blinded research assistants, reducing the risk of measurement bias.

Despite these strengths, several limitations should be considered. First, primary dropouts were observed, particularly due to a lack of interest or time. A mismatch was observed between the study’s health-promotion and prevention focus and the self-perception of the target group. Many older persons did not identify as being at risk or in need of health-promoting or preventive interventions, which may have affected both recruitment and engagement. As a result, the generalisability of the findings may be limited, and there is a risk of selection bias, as participants may have been more health-conscious or motivated than the general population.

Second, a limitation relates to the sample characteristics. The strict inclusion criteria resulted in a relatively healthy and homogeneous group, which strengthened internal validity by reducing confounding factors. However, this also limits external validity, as the findings may not be generalisable to more frail older persons or those with multiple chronic conditions. In addition, participants were recruited from two urban districts with slightly higher socioeconomic status than the general population, which may have influenced both participation and outcomes. These factors should be considered when interpreting the results, as they suggest that the observed effects may represent an upper bound of what can be achieved in healthier populations. Future research should explore whether similar interventions are effective among more diverse and vulnerable groups, including those with lower socioeconomic status, multimorbidity, or living in rural areas, to ensure broader applicability and equity in health-promotion strategies.

Third, although all assessors were trained and inter-rater reliability was tested, the use of professionals from different backgrounds (e.g., occupational therapists, physiotherapists, registered nurses) may have introduced variability in measurement quality.

Fourth, a methodological consideration concerns the handling of missing data. Although multiple imputation is commonly applied in RCTs, the MCD method was considered more appropriate for this study population. This approach provides a transparent, non-parametric, and conservative imputation strategy that avoids strong distributional assumptions.

Fifth, several leisure outcomes were evaluated across intervention groups and follow-up time points without formal adjustment for multiple comparisons. This may increase the risk of type 1 error, and some statistically significant findings may therefore represent chance findings. The results should therefore be interpreted with caution and viewed primarily as indicators of potential patterns in leisure participation that warrant further investigation.

Sixth, an additional consideration is that the trial was conducted between 2007 and 2011, more than 15 years ago. During this period, healthcare systems, digital technologies, and models of service delivery have evolved, and societal patterns of leisure participation among older persons may also have changed. For example, digital forms of social and cultural engagement have become more widespread, potentially influencing both opportunities for participation and barriers to access. Although service organisation and digital infrastructures have developed substantially, these developments have not uniformly resulted in improved access to, or availability of, services for older persons. In several contexts, organisational pressures, resource constraints, and increased system demands remain or have intensified. Thus, while the specific context in which the interventions were delivered differs from today’s environment, the underlying aims of promoting participation and preventing functional decline remain highly relevant. This temporal gap should nevertheless be considered when interpreting the findings and assessing their transferability to contemporary settings. At the same time, the long-term follow-up and sustained relevance of the findings suggest that the underlying mechanisms linking health-promotion and leisure participation may be robust across changing societal contexts.

Finally, while the interventions showed positive effects on social and hobby-related activities, no statistically significant effects were found for cultural or physical activities over the 24-month period. This may indicate that these domains require more person-centred and targeted health-promoting strategies. The lack of sustained impact in these areas highlights a potential gap in the intervention design and suggests the need for future studies to explore tailored approaches that better address individual preferences and barriers to participation in cultural and physical activities. 

## Conclusion

This study demonstrates that health-promotion interventions, particularly structured and group-based formats like senior meetings, can play a critical role sustaining participation in leisure activities among older persons. By supporting continued participation in these activities, these interventions may contribute to healthier, more fulfilling ageing. Providing information, encouraging positive attitudes, and facilitating open discussions about leisure activities appear to be important for effective health-promotion. Tailoring support to individual preferences and motivations, especially as older persons adapt to changes in how they participate in activities, could be key to maintaining participation and promoting long-term well-being.

## Data Availability

The dataset supporting the findings of this study are available, but restrictions apply to the availability of these data due to ethical requirements and so are not publicly available. Data are however available from the researcher responsible for keeping the dataset upon reasonable request. As this person may change over time, please enquire the responsible researcher’s contact information at [https://researchdata.se/en](https:/researchdata.se/en) , entering the search string ”Elderly persons in the risk zone”.

## Abbreviations

MMSE: Mini Mental State Examination
MNPS: Modified NPS Interest Checklist
NPS: Norling, Pettersson, Selander
MCD: Median Change of Deterioration
RCT: Randomised Controlled Trial
ITT: Intention To Treat
